# Orofacial Neuropathic Pain Leads to a Hyporesponsive Barrel Cortex with Enhanced Structural Synaptic Plasticity

**DOI:** 10.1371/journal.pone.0160786

**Published:** 2016-08-22

**Authors:** Karine Thibault, Sébastien Rivière, Zsolt Lenkei, Isabelle Férézou, Sophie Pezet

**Affiliations:** 1 Brain Plasticity Unit, ESPCI, PSL Research University, 10 rue Vauquelin, 75005, Paris, France; 2 Centre National de la Recherche Scientifique, UMR 8249, 75005, Paris, France; University of Toronto, CANADA

## Abstract

Chronic pain is a long-lasting debilitating condition that is particularly difficult to treat due to the lack of identified underlying mechanisms. Although several key contributing processes have been described at the level of the spinal cord, very few studies have investigated the supraspinal mechanisms underlying chronic pain. Using a combination of approaches (cortical intrinsic imaging, immunohistochemical and behavioural analysis), our study aimed to decipher the nature of functional and structural changes in a mouse model of orofacial neuropathic pain, focusing on cortical areas involved in various pain components. Our results show that chronic neuropathic orofacial pain is associated with decreased haemodynamic responsiveness to whisker stimulation in the barrel field cortex. This reduced functional activation is likely due to the increased basal neuronal activity (measured indirectly using cFos and phospho-ERK immunoreactivity) observed in several cortical areas, including the contralateral barrel field, motor and cingulate cortices. In the same animals, immunohistochemical analysis of markers for active pre- or postsynaptic elements (Piccolo and phospho-Cofilin, respectively) revealed an increased immunofluorescence in deep cortical layers of the contralateral barrel field, motor and cingulate cortices. These results suggest that long-lasting orofacial neuropathic pain is associated with exacerbated neuronal activity and synaptic plasticity at the cortical level.

## Introduction

Trigeminal neuralgia is the most common orofacial neuropathic pain disorder [1,2]. This debilitating disorder is often manifested with thermal and mechanical allodynia and hyperalgesia [1]. Chronic orofacial pain disorders, among various other conditions (*e*.*g*. temporomandibular disorders), can be difficult to treat due to a maladaptive plasticity that is not well understood [3]. Fundamental research has revealed several key mechanisms underlying neuropathic pain at the level of the spinal cord, such as decreased inhibition [4], glial activation through a BDNF-dependent mechanism [5], enhanced release of cytokines and chemokines [6] and increased receptor activation [3][7,8]. By contrast, far less is known about the supraspinal changes underlying neuropathic pain. Clinical studies have reported strong cortical rearrangements in the primary somatosensory cortex in several neuropathic pain conditions including phantom limb pain [9], chronic back pain [10], fibromyalgia [11], complex regional pain syndrome (CRPS) [12,13][14], unilateral chronic pain [15] and chronic back pain [16](see [17] for review). Strikingly, two separate teams have shown that different chronic pain diseases (*e*.*g*. chronic back pain, CRPS and knee osteoarthritis [18]) exhibit unique anatomical ‘brain signatures’ [18,19], *i*.*e*. these specific anatomical brain reorganizations may reflect a unique maladaptive physiology in these different types of chronic pain. Interestingly, this cortical plasticity is reversed by treatment coincident with clinical improvement [14]. Maladaptive changes in the primary and secondary somatosensory cortices have also been demonstrated in chronic orofacial pain syndromes, including trigeminal neuropathic pain and temporomandibular joint disorder pain [20] (and see [19] for a meta-analysis of both syndromes). Other brain areas comprising the anterior cingulate/mid-cingulate cortex and prefrontal cortex (such as the thalamus or cortical areas involved in the emotional aspects of pain) also display modified activation patterns [19]. Global structural changes, including modifications in grey matter volume [21,22], white matter density [23,24] and fractional anisotropy [22,23] have also been reported. Furthermore, subtle anatomical changes in the prefrontal cortex of patients with chronic orofacial pain can induce changes in their personalities [25,26]. However, while these studies suggest important structural plasticity of cortical regions in chronic pain patients, the underlying molecular and functional changes are still poorly understood. Notably the link between the cortical responsiveness to stimulus application and the concomitant level of neuronal activity and structural synaptic changes in these brain areas is unclear.

To study orofacial neuropathic pain, a model of the chronic constriction injury (CCI) model initially developed by Bennett and Xie in rats at the level of the sciatic nerve [27] has been modified [28]. This approach exploits the rodent trigeminal nervous system, which is anatomically composed of four nuclei (the mesencephalic trigeminal nucleus, motor trigeminal nucleus, principal trigeminal nucleus, and spinal trigeminal nucleus) and the trigeminal ganglion (TG). The spinal trigeminal nucleus caudalis (Sp5C) is a critical relay site for processing nociceptive afferent input from the orofacial area, and for modulating neuroplastic changes [29]. The infraorbital nerve (ION), which is solely composed of sensory fibres, nearly forms the entire maxillary division of the trigeminal nerve in rats. CCI of the ION (CCI-ION) therefore provides a relevant experimental rat model of trigeminal neuropathic pain [28,30,31].

The aim of this study was to determine the possible functional and molecular mechanisms taking place in several cortical areas in neuropathic pain, using a mouse model of ION ligation. Functional intrinsic imaging of the primary sensory cortex barrel field (S1BF) has allowed us to demonstrate a decreased evoked response to whisker stimulation that is due to an enhanced baseline neuronal activity. Immunohistochemical analysis revealed that the primary sensory, motor and cingulate cortices are all subject to strong pre- and postsynaptic changes. These results provide a basis for a better understanding and possible new therapeutical strategies of orofacial neuropathic pain.

## Materials and Methods

### Animals

30 male C57/BL6 mice (Janvier, France) were used in this study (n = 12 mice, including n = 6 mice with ligation of the infraorbitrary nerve (ION) and n = 6 sham operated mice, for the longitudinal behavioural, imaging and immunohistochemical study, and n = 18 additional mice for the c-fos experiment, see below). Mice were 4 weeks old at the beginning of the experiments. All experiments were performed in agreement with the European Community Council Directive of September 22^nd^, 2010 (010/63/UE) and the local ethics committee (*Comité d’éthique en matière d’expérimentation animale n°59*, *C2EA –59*, *‘Paris Centre et Sud’*). The project was accepted by this ethics committee under the reference #A-1722. The guidelines for investigating experimental pain in animals published by the International Association for the Study of Pain (IASP) were followed. Great care was taken, particularly regarding housing conditions, to avoid or minimize discomfort of the animals. Mice were housed in a solid-floor cage with food and water available *ad libitum* and a deep layer of sawdust to accommodate excess urine. The cages were changed twice a week and kept at a constant temperature (22 ± 1°C) with a 12-hour alternating light/dark cycle.

### Surgical implants

Thirty days before the infraorbital nerve (ION) ligation, 12 mice were implanted with a head-fixation post and an imaging chamber to allow subsequent repeated imaging sessions. The animals were anesthetized with a mixture of medetomidine hydrochloride (0.8 mg/kg) and ketamine (60 mg/kg) and maintained at 37°C with a heating blanket. Several minutes after a subcutaneous injection of lidocaine (10 mg/ml) to the head of the animal, the skin covering the interparietal and right parietal bones of the skull was excised. Following a careful cleaning, the exposed intact skull was protected with a layer of cyanoacrylate glue. A metal head-fixation post was then glued on to the interparietal bone and a circular plastic chamber (~6 mm in diameter and 1 mm thick) was glued on to the right parietal bone (the centre of the chamber was located at 1.5 mm posterior to the bregma, and 3.3 mm to the right of the midline). The head post and chamber fixations were secured with dental cement. The anaesthesia was reversed at the end of the implantation procedure by a subcutaneous injection of atipamezole (50 μg).

### Habituation to the orofacial stimulation test

After at least 5 days of recovery, the animals (n = 12) were subjected to a habituation period (S1 Table). Mice were first habituated to the room, the experimenter, and then to the orofacial stimulation test (mechanical orofacial test; Ugo Basile, Italy). The orofacial stimulation test measures hypersensitivity to mechanical stimulation of the trigeminal area. Mice were allowed to voluntarily contact a mechanical stimulator (consisting of metal hairs attached to a mounting plate) with their vibrissal pad in order to access a sweet water reward (S1 Table). Drinking duration was measured by detecting the interruption of an infrared beam traversing the opening to the reward. There was no mechanical stimulation at the beginning of the habituation period, and the mice could access the sweetened water just by inserting their noses into the reward opening.

Mice were water-deprived for the subsequent habituation sessions (*i*.*e*. the water dispenser was removed from the cages 12 hours before the behaviour session) and the mechanical stimulation was progressively added by increasing the number of metallic hairs attached to the mounting plate (from 12 to 18).

During the first week of habituation, the mice were habituated (without being water-deprived) 3 at a time, twice a week in the apparatus, without any metal hair. During the second week of habituation (« -3 » in S1 Table), the mice were water-deprived and habituated 3 at a time, twice a week in the apparatus, without any metal hair. We had to habituate them by 3 initially because we experienced previously that some mice learn slowly to get the sweet water from the drinking port. By habituating initially the mice by groups of three, we did not observe as much "slow learners", probably since they could watch the other mice drinking.

The third week of habituation, the mice were habituated individually in the apparatus, one session without hair, then one session with 12 hairs and finally three sessions with 18 hairs (i.e. 5 sessions in the week). The fourth week of habituation, the mice went through one last individual session with 18 hairs.

### Behavioural test: Assessment of mechanical sensitivity

The orofacial stimulation test was used to measure the sensitivity threshold of the 12 mice. Mechanical sensitivity was defined as a significant decrease in drinking duration. Mice were tested after 12 hours of water deprivation using a mechanical stimulator consisting of 18 metallic wires. The baseline was measured one week before the ION ligation (S1 Table). The baseline drinking duration percentage was calculated using individual values as follows: (W/BL)X100 where *W* corresponds to the week of interest: 1W, 2W, 3W, 4W and 5W. Results are expressed as the baseline percentage ± SEM. Animals that were not engaged in drinking at the end of the baseline session were excluded from the study (see S1 Table). Although we began with n = 6 animals per experimental group, the final number of animals included per group was n = 4 (see S1 Table).

### Ligation of the infraorbital nerve (ION)

Mice were anesthetized with a mixture of medetomidine hydrochloride (0.8 mg/kg) and ketamine (60 mg/kg) and maintained at 37°C with a heating blanket. Unilateral ligation to the left ION was performed as previously described [29]. An incision was made along the left cheek to expose the ION where it emerges from the infraorbital fissure [30]. After local administration of lidocaine (10 mg/ml), the ION was gently isolated using forceps. Two tight ligations of the nerve (~1 mm from each other) were made with 5.0 diameter catgut sutures (Chromic gut, 5–0; ref. 1248, Angiotech Pharmaceuticals; Canada), which constricted the nerve to approximately 1/3 to 1/2 of its initial diameter [24]. The incision was closed using silk sutures. A subcutaneaous injection of Metacam (1 mg/kg) was performed in order to prevent postsurgical pain. For the sham-operated mice, the ION was exposed on the left side using the same procedure, although the nerve was not ligated.

### Optical imaging of intrinsic signals: Assessment of cortical responses to tactile stimuli

For each imaging session, implanted animals were anesthetised with a mixture of medetomidine hydrochloride (0.8 mg/kg) and ketamine (60 mg/kg) and maintained at 37°C with a heating blanket. The cortex was illuminated at 570 nm, through the exposed intact bone within the implanted imaging chamber. The reflected light was collected at 20 Hz through a tandem lens microscope onto a CMOS camera (MiCAM Ultima, SciMedia; Japan). The camera’s 10,000-pixel sensor covered a 10 x 10 mm field of view, providing a resolution of 100 μm per pixel. Relative variation of the reflected light was measured in response to left single whisker deflections (100 Hz for 1 s) and averaged over 100 trials. This intrinsic imaging protocol was run successively for each mouse at each imaging session, using two different whiskers (typically A1 and C1, although B1 and C1 were used for one mouse that lacked A1). These imaging sessions were performed weekly for each animal, starting before the ION ligation (BL) and running until 4 weeks after BL. Results are expressed as (R-R_0_)/R_0_, with R representing the averaged reflected light measured over 1 s immediately after the stimulus presentation and R_0_ being the average reflected light measured over 1 s before the stimulus presentation Images obtained from such computation indeed show the spatial extent of the haemodynamic response evoked by the whisker stimulation. Spatial profiles of reflected light were computed from these images along the rostro-caudal axis. Minimal values of these profiles were used to quantify the response amplitude for each condition.

The results from several mice had to be discarded, as they lost some of their whiskers during the 4 weeks of longitudinal follow-up. Consequently, the final number of animals included in each experimental group was n = 3 instead of the initially planned n = 6. A table presented in S1 Table is showing the time points at which animals were excluded and why. Nevertheless, two whiskers were tested independently for each mouse on each run and these results were considered as independent, yielding n = 6 tests of cortical functional activation induced by whisker stimulation per experimental group.

### Design of the cFos experiment

In order to investigate if the S1BF of ION mice was subjected to an increased neuronal activity that would unable the possible induction of further neuronal activation following whisker stimulation, we quantified the number of c-fos positive profiles in the S1BF submitted (or not) to sustained whisker activation in freely moving animals. To do so, we used a classical approach that involves the clipping of all whiskers except one or one raw [32,33]. The animal is placed for two hours in a dark environment where it can explore various objects, leading to a sustained sensory activation. In details: eighteen animals were used in total, randomised in 4 experimental groups: two ION groups of animals with ION ligations (n = 9), and two sham groups of control animals (n = 9). Mice from the first ION (n = 5) and sham (n = 4) groups were anesthetized with isoflurane (induction: 3–4%, sustaining: 1.5%) and all but one row of whiskers (row C) on the left side were clipped close to the skin. The whiskers on the right side were left intact. Animals were placed by pairs (when possible) in a stimulating box that was furnished with various objects in order to provide a tactile-enriched environment for two hours in the dark. After the exploration period, animals were anesthetized with urethane (1.75 g/kg i.p.) and perfused. The remaining ION (n = 4) and sham (n = 5) groups were removed from their home cages and immediately anesthetized and perfused for use as controls to obtain a basal level of cFos expression.

### Immunohistochemistry

A day after the end of the last imaging session, animals were deeply anaesthetized *via* urethane injection (1.75 g/kg i.p) and transcardially perfused with 10 ml of 0.9% NaCl, followed by 50 ml of 4% paraformaldehyde (PFA) plus 15% picric acid in 0.2 M phosphate buffer (PB), pH 7.4. Urethane was used here for the perfusion, according to a previously described procedure for the immunostaining of p-Erk in pain pathways [34]. TGs and the brain were dissected out and post-fixed in 4% PFA plus 15% picric acid for 2 hours at room temperature or overnight at 4°C, respectively. The TG and the brain were then cryoprotected in 30% sucrose in 0.2 M PB (pH 7.4) overnight at 4°C, and were subsequently cut into a series of 14-μm longitudinal (TG) or 20-μm coronal (brain) serial sections with a cryostat (HM550, Microm Microtech).

### ATF3 immunostaining in the TG

In order to assess that all animals had the same level of nerve injury, we quantified the percentage of ATF3 positive cells in the DRG. Indeed, ATF3 has been previously shown to be overexpressed in lesioned DRG neurons [35]. Non-lesioned neurons do not express this transcription factor [35]. Serial sections from the ipsilateral and contralateral TGs were incubated overnight at room temperature with a mixture of the antibodies ATF3 (1:500 rabbit anti-ATF3 (C19); ref. sc-188, Santa Cruz Biotechnology; USA) and βIII tubulin, a neuronal marker, (1:2000 mouse anti-βIII Tubulin; ref. G7121, Promega; San Luis Obispo, CA, USA) diluted in PBS-T-azide (0.02M saline PB containing 0.3% Triton X-100 and 0.02% sodium azide). TG sections were then washed in PBS and subsequently incubated for 2 h at room temperature in 1:1000 Alexa Fluor® 488 anti-rabbit IgG and Alexa Fluor® 350 anti-mouse IgG (Invitrogen; California, USA). Sections were finally washed in PBS and mounted in Vectashield medium (Vector; Burlingame, USA).

### Quantification of ATF3- and βIII tubulin-positive neurons in the TG

The proportion of ATF3- and βIII tubulin-expressing TG cells was quantified by counting the number of positive cells with visible nuclei in images acquired using a 40x objective. All acquisitions were performed using the same settings. To determine the total number of neuronal cells, βIII tubulin staining was used as a neuronal marker to manually count the cells with visible nuclei. At least 200 cells (across four or five sections) were counted per TG. Results are expressed as the percentage of ATF3-positive cells out of the total number of cells in the TG.

### cFos and p-Erk immunostainings in the brain

Serial sections from the brain were immunostained for cFos according to the avidin-biotin-peroxidase method and followed by DAB revelation. Tissue sections were incubated overnight at room temperature in primary antiserum (diluted in PBS-T-azide) directed against either the cFos protein (1:5000 rabbit anti-cFos; ref. sc-52, Santa Cruz Biotechnology; USA) or against the p-Erk protein (1:1000 rabbit anti-phospho p44/42 MAPK; ref. 4370L, Cell Signaling). Incubated sections were washed in three successive baths of PBS and subsequently incubated in 1:300 biotinylated goat anti-rabbit IgG (Vector; Burlingame, USA) in PBS-T for 1 h at room temperature. Sections were then washed three times in PBS and incubated for 1 h in avidin-biotin-peroxidase complex (Vectastain, Vector Laboratories) diluted 1:500 in PBS. Finally, after 3 washes in PBS, staining was revealed with a peroxidase substrate kit (Vector Laboratories, SK-4100) according to the manufacturer’s protocol. The reaction proceeded at 20°C under the visual control of a light microscope, and was stopped 5 min later by washing in distilled water. Sections were sequentially dehydrated through an alcohol series (70%, 90%, 95% and absolute alcohol), air-dried, xylene treated, and cover-slipped with DPX mounting medium for histology (Sigma). Brain sections of mice from all experimental groups were simultaneously immunostained to accommodate quantification of the number of immunoreactive neurons and to correct for immunochemistry variation between experiments.

### Quantification of cFos-positive neurons

Tissue sections were first examined using dark-field microscopy to determine structures of interest. To quantify cFos staining in both groups of animals, black and white images with 256 grey levels were acquired with a 5x objective on a Zeiss microscope. All acquisitions were performed with the same acquisition settings. We quantified cFos positive neurons in the S1BF cortex contralateral to the ION ligation. To count the number of nuclei, we performed a particle analysis using the ImageJ software (USA). Briefly, a threshold is set and pixels with values within this range are converted to black; pixels with values outside of this range are converted to white. The threshold was determined to obtain clear objects representing cFos-labelled nuclei. The same threshold was applied to each image for all animal groups. Only objects with an area superior to 25 μm^2^ were counted.

### Quantification of p-Erk-positive cells

Tissue sections were first examined using dark-field microscopy to determine structures of interest. Three specific regions were defined (the S1BF cortex, the motor cortex including primary and secondary motor cortices, and the cingulate cortex) and the number of p-Erk-positive cells in each area was counted. All p-Erk immunoreactive cells were counted regardless of staining intensity. The investigator responsible for counting p-Erk-positive cells was blind to the animal’s treatment.

### Piccolo and p-Cofilin immunostainings in the brain

Serial sections from the brain were incubated with either anti-Piccolo (1:100; rabbit, ref. 20664, Abcam; Cambridge, UK) or anti-p-Cofilin protein (1:300; rabbit, ref. 47281, Abcam; Cambridge, UK) as primary antibodies in PBS-T-azide overnight at room temperature. After washings in PBS, the sections were incubated in Alexa Fluor® 488 anti-rabbit IgG (1:1000; Invitrogen; California, USA) in PBS-T for 2 h at room temperature. Following PBS washes, the sections were mounted in Vectashield medium (Vector Laboratories; Burlingame, USA).

### p-Cofilin/NeuN double staining in the brain

Serial brain sections were incubated overnight at room temperature with a mixture of the antibodies anti-p-Cofilin (1:300; rabbit, ref. 47281, Abcam; Cambridge, UK) and the neuronal marker anti-NeuN (1:1000 mouse anti-NeuN; ref. VMA 377, Abcys) diluted in PBS-T-azide. Following washes, sections were incubated 2 h in Alexa Fluor® 568 anti-rabbit IgG and Alexa Fluor® 488 anti-mouse IgG (Invitrogen; California, USA) diluted 1:1000 in PBS-T. Sections were washed and mounted in Vectashield medium.

### Quantification of Piccolo staining

Analysis was performed on the contralateral side of the brain relative to the ION ligation. To quantify Piccolo staining in both groups of animals, black and white images with 256 grey levels were acquired with a 20x objective on a Zeiss microscope. All acquisitions were performed with the same acquisition settings. To quantify the staining, we performed an area fraction analysis using the ImageJ software (USA). Briefly, a threshold is set and pixels with values within this range are converted to black; pixels with values outside of this range are converted to white. The threshold was determined to obtain a clear area representing Piccolo labelling. This analysis measured the area of labelling in the previously described regions of interest (the S1BF, motor and cingulate cortices). Area fraction, representing the percentage of pixels in the selection that were highlighted in black, was compared between sham and ION-ligatured animals. Quantification was performed on 4 sections for each animal using the following bregma coordinates: 0.36 mm, -0.46 mm, -1.22 mm and -1.94 mm. For each cortical region, quantification was performed within two regions of interest (325 x 440 μm): one located on the superficial laminae (II-IV) and the other located on the deep laminae (V and VI).

### Quantification of p-Cofilin staining

The contralateral side of the brain relative to the ION ligation was analysed. To quantify p-Cofilin staining in both groups of animals, black and white images with 256 grey levels were acquired with a 20x objective on a Zeiss microscope. All acquisitions were performed with the same acquisition settings. We focused our quantification on the number of neurons displaying at least 5 punctate labellings in their cell bodies. A double labelling with anti-NeuN was used to facilitate quantification. The number of p-Cofilin-positive neurons was counted for each specific cortical area. Quantification was performed in the same coordinates as those used for Piccolo staining quantification, and in the same regions of interest (325 x 440 μm). The investigator responsible for the counting of p-Cofilin-positive neurons was blind to the animal’s treatment.

### Data analysis

#### Behavioural experiments

Mechanical sensitivity results were analysed by two-way analysis of variance (ANOVA) followed by a Student-Newman-Keuls post-hoc test for multiple comparisons using the SigmaStat software. ANOVA results are reported as the value of the Fisher distribution *Fx*,*y* obtained from the data, where *x* is the degrees of freedom for groups and *y* is the total degrees of freedom of the distribution.

#### Imaging experiments

Responses to tactile stimuli were compared between the experimental groups throughout the imaging sessions, using two-way analysis of variance (ANOVA) followed by a Student-Newman-Keuls post-hoc test (SigmaStat).

#### Immunohistochemistry experiments

Quantification of ATF3+ neurons was evaluated by two-way analysis of variance (ANOVA), using the treatment (sham or ION) and the side analysed (ipsilateral or contralateral) as the two variables. Two-way ANOVA was performed to analyse the cFos experiments using the treatment (sham or ION) and the stimulations (after stimulations or no stimulation) as the two variables. Two-way ANOVA was performed to quantify p-Erk+ cells using the treatment (sham or ION) and the cortical area analysed (barrel, motor or cingulate cortex) as the two variables. Two-way ANOVA tests were followed by a Student-Newman-Keuls post-hoc test for multiple comparisons (SigmaStat). Finally, three-way ANOVA tests were performed to quantify Piccolo and p-Cofilin staining, using the treatment (sham or ION), the cortical area analysed (S1BF, motor cortex or cingulate cortex) and the analysed cortical depth (superficial or deep) as variables. The three-way ANOVA was followed by a Student-Newman-Keuls post-hoc test for multiple comparisons. ANOVA results are reported as the value of the Fisher distribution *Fx*,*y* obtained from the data, where *x* is the degrees of freedom for groups and *y* is the total degrees of freedom of the distribution. Data were expressed as means ± standard error of the mean (SEM), with the following levels of significance: *p<0.05, ** p<0.01, and ***p<0.001. For all quantifications of the immunostainings, coordinates and delinations of the brain areas were taken from [36,37]. Quantifications performed at the level of the cingulate cortex were taken in the anterior cingulate cortex; more precisely in both the Cg2 and Cg1 also, denominated 24b and 24a, respectively by Vogt and Paxinos [37]. Note that a mini dataset (S1 Dataset) is providing individual results.

## Results

### Changes in sensitivity and cortical activity in the primary somatosensory cortex following unilateral ION ligation

**Fig 1:**
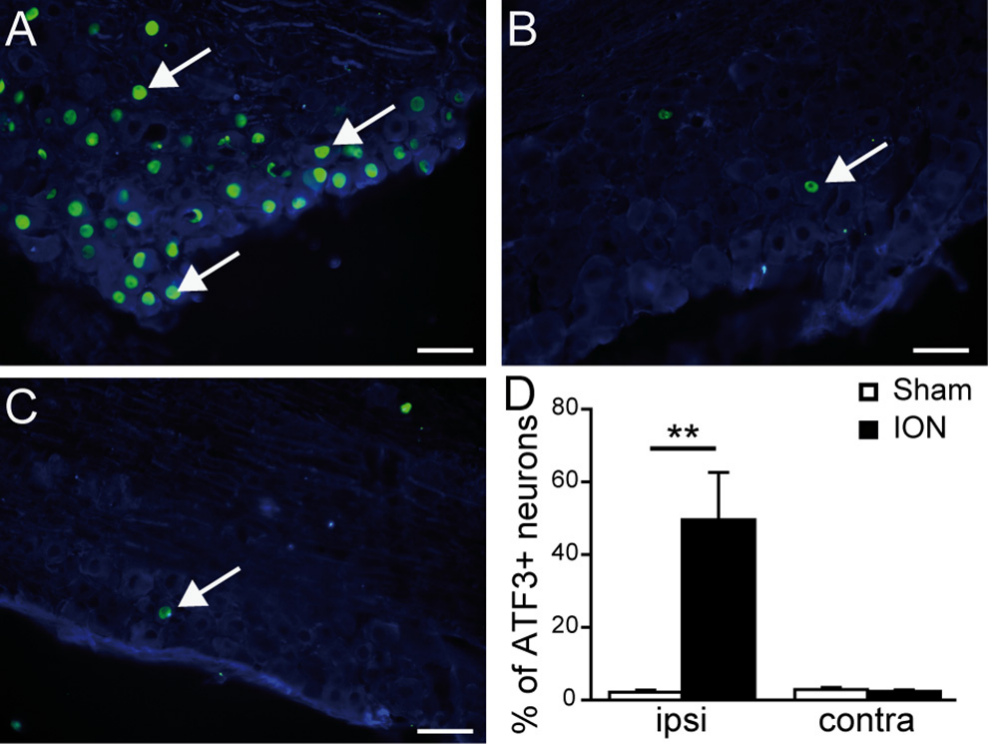
Upregulation of ATF3 in sensory neurons of the trigeminal ganglion after ION ligation. A-C: Double immunostaining showing ATF3 (green)/βIII tubulin (blue) positive neurons in the ipsilateral (A) and contralateral TG (B) of a representative ION animal, and the ipsilateral TG of a sham animal (C). D: Quantification of the percentage of ATF3-positive neurons in sham and ION (animals that received a ligation of the Infraorbitrary nerve) mice, 5 weeks after surgery, in the ipsilateral (ipsi) and contralateral (contra) sides of the lesion. Nerve ligation induced a statistically significant upregulation of ATF3 in the ipsilateral TG of ION animals (F_1,21_ = 15.927; p<0.001). Scale bars = 50μm. ** p<0.01 ION *vs*. sham animals (n = 6 sham, n = 5 ION). Data are expressed as means ± SEM.

We focused on the possible functional and molecular mechanisms following peripheral nerve injury. ATF3 has previously been used as a marker for injured neurons in the dorsal root ganglion after peripheral nerve injury [35]. Here, in order to verify that all the animals included in this study that underwent ION ligation had the same extent of nerve lesion, we analyzed ATF3 expression in the TGs. Five weeks after the surgery, we observed a significant increase in ATF3 expression specifically in the ipsilateral TG of ligated mice (Fig 1A and 1D; p<0.001). In contrast, no significant ATF3 expression was found in the contralateral TG of ION mice (Fig 1B and 1D) or in the ipsilateral TG of sham-operated mice (Fig 1C and 1D).

**Fig 2:**
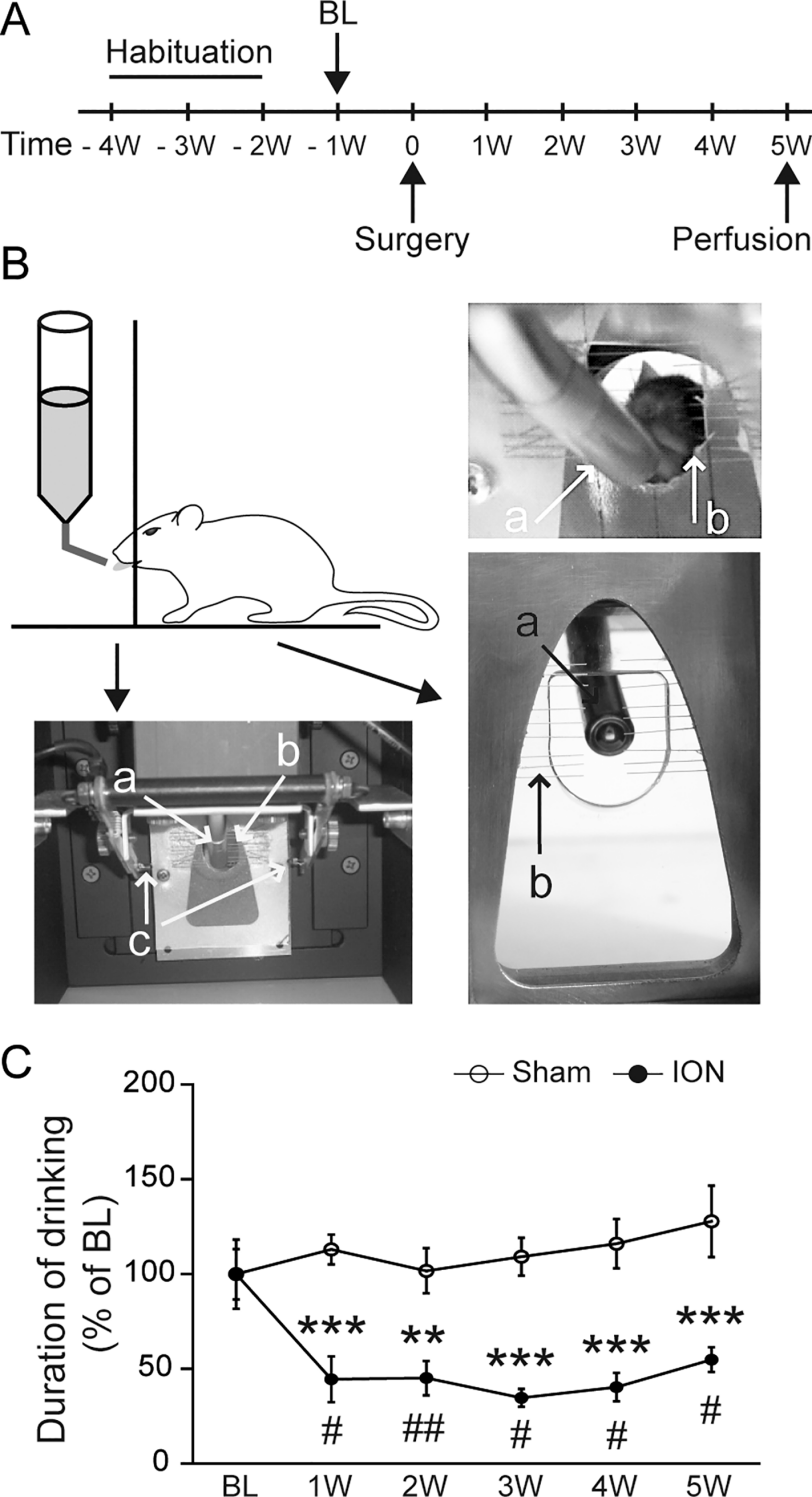
ION ligation induces mechanical hyperalgesia. A: Schematic representation of the experimental paradigm. Habituation to the test lasted 3 weeks, and the baseline (BL) values were measured 1 week before the surgery (-1W). Subsequently, mice were tested once per week. B: The orofacial stimulation test. In this paradigm, mice were allowed to voluntarily contact a mechanical stimulator (b) in order to access a sweet water reward (a). Automatic quantification of the drinking duration was performed using an infrared beam traversing the opening to the reward (c). C: ION ligation induced mechanical hyperalgesia (F_1,44_ = 63.249; p<0.001). The drinking duration decreased during the first week after surgery (1W) as compared to the BL, and persisted for 5 weeks. **p<0.01 and ***p<0.001 *vs*. sham mice; #p<0.05 and ##p<0.01 *vs*. BL (n = 4 sham, n = 4 ION). All data are expressed as means ± SEM.

In order to measure the changes in orofacial sensitivity evoked by the unilateral ION ligation, we used an operant test in which a mildly water-deprived mouse will voluntarily approach and drink a sweet water reward by introducing its snout in an apparatus that provides mechanical stimulations to the whisker pad (Fig 2). This paradigm involves a conflict between the willingness to drink and the perceived discomfort/pain due to this mechanical stimulation. The time spent drinking was quantified by using an infrared beam. ION-ligated mice displayed a significant decrease in drinking duration as compared to the sham-operated animals, as early as 1 week after the surgery (Fig 2C; p<0.05). This decrease continued for 5 weeks (Fig 2C; p<0.001), suggesting the persistence of mechanical orofacial allodynia.

In order to decipher the link between the alteration of functional plasticity of the barrel field of the primary sensory cortex and mechanical allodynia in this model, we carried out a longitudinal study of the haemodynamic responses induced by whisker stimulations in the S1BF cortex under anaesthesia.

Indeed, focal cortical activation is known to induce a local increase of cerebral blood flow and volume (so called haemodynamic response), in addition to changes in haemoglobin concentration and oxygenation (metabolic response). Optical imaging of intrinsic signals consists in monitoring changes in light reflectance of the cortical surface resulting from these local physiological correlates of neuronal activity. When using light at 570 nm, which corresponds to an isosbestic point at which oxyhaemoglobin and deoxyhaemoglobin share the same absorption properties, the variations of light absorption by the cortical surface reflect changes in blood volume [38]. It has been shown in the rat barrel cortex that this measure of light reflectance at 570 nm is an efficient way to visualise the cortical activation evoked by a single whisker deflection [39]. Indeed, the observed decrease in reflected light following tactile stimulation is linearly correlated with the integrated measure of extracellular field potentials [39,40].

**Fig 3:**
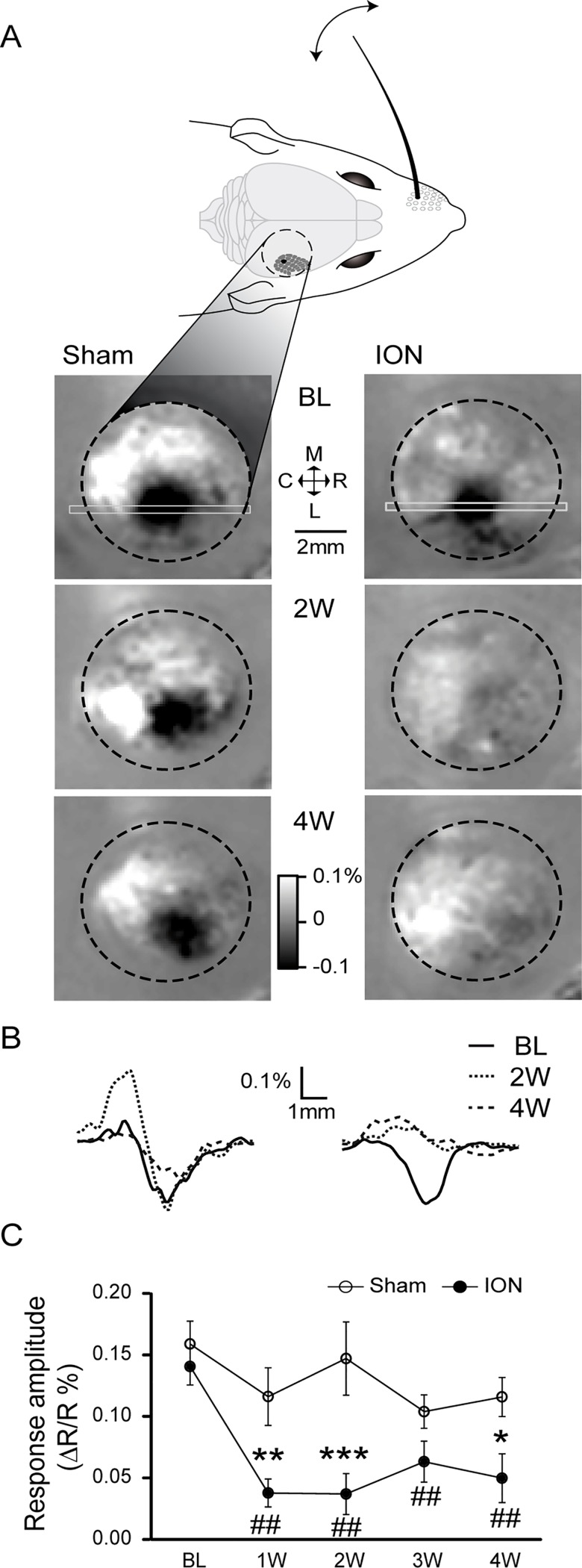
Intrinsic optical imaging reveals a decrease in sensory-evoked signals in the contralateral somatosensory cortex after ION ligation. A: Intrinsic optical signals were recorded through the intact skull, on the right posteromedial barrel subfield of the primary omatosensory cortex (S1BF; the black dotted line designates the borders of the imaging chamber). Variation in the reflected light (at 570 nm) induced by repetitive C1 whisker deflections (1 s at 100 Hz) is shown for a sham-operated (left) and an ION-ligated mouse (right). The results obtained before surgery (Baseline: BL, upper images) or 2 and 4 weeks after the surgery (2W and 4W, respectively, lower images) are expressed as R-R_0_/R_0_, with R representing the averaged reflected light measured over 1 s immediately after the stimulus presentation and R_0_ representing the reflected light averaged over 1 s before the stimulus presentation. B: Spatial profiles of the reflected light measured along the rostro-caudal axis, using the region of interest indicated in A by the light grey rectangle. Note the absence of signal variation for the ION-ligated mouse at 2W and 4W. C: Quantification of the evoked signals was performed by looking for the minimal values of the spatial profiles (with examples shown in B). The ION ligation resulted in a significant decrease in evoked signals that persisted from the first week after the surgery through the following weeks. *p<0.05 and **p<0.01 ***p<0.001 *vs*. sham mice; #p<0.05 and ##p<0.01 *vs*. BL (n = 6 sham, n = 6 ION). All data are expressed as means ± SEM.

We chose to use this intrinsic imaging approach to study the functional cortical plasticity since it can be performed through the intact skull in mice, keeping the brain’s entire integrity [39]. We were indeed able to repeatedly image cortical activations evoked by repeated whisker deflections (Fig 3), with the same groups of mice (ION n = 6 datasets, sham n = 6 datasets, see Materials and Methods section). Although the evoked optical signals measured in sham-operated animals were stable over time, we observed a rapid decrease in the cortical responses imaged in the ION-ligated mice, which was statistically significant as early as one week after the surgery (Fig 3C).

We were initially surprised to observe this decreased sensory response concomitant with the appearance of mechanical hyperalgesia in the ION model (Fig 2C), since an increased local neuronal responsiveness to hindpaw stimulation was previously observed in the rat primary sensory cortex in models of sciatic nerve lesion, both with voltage-sensitive dye imaging [41] and indirectly by fMRI [42]. One possible explanation is that the measured decrease in responses could partially result from an increase in the basal level of neuronal activity, since intrinsic imaging measures the relative changes in cerebral blood volume during the whisker stimulation over the initial basal level (before stimulation).

**Fig 4:**
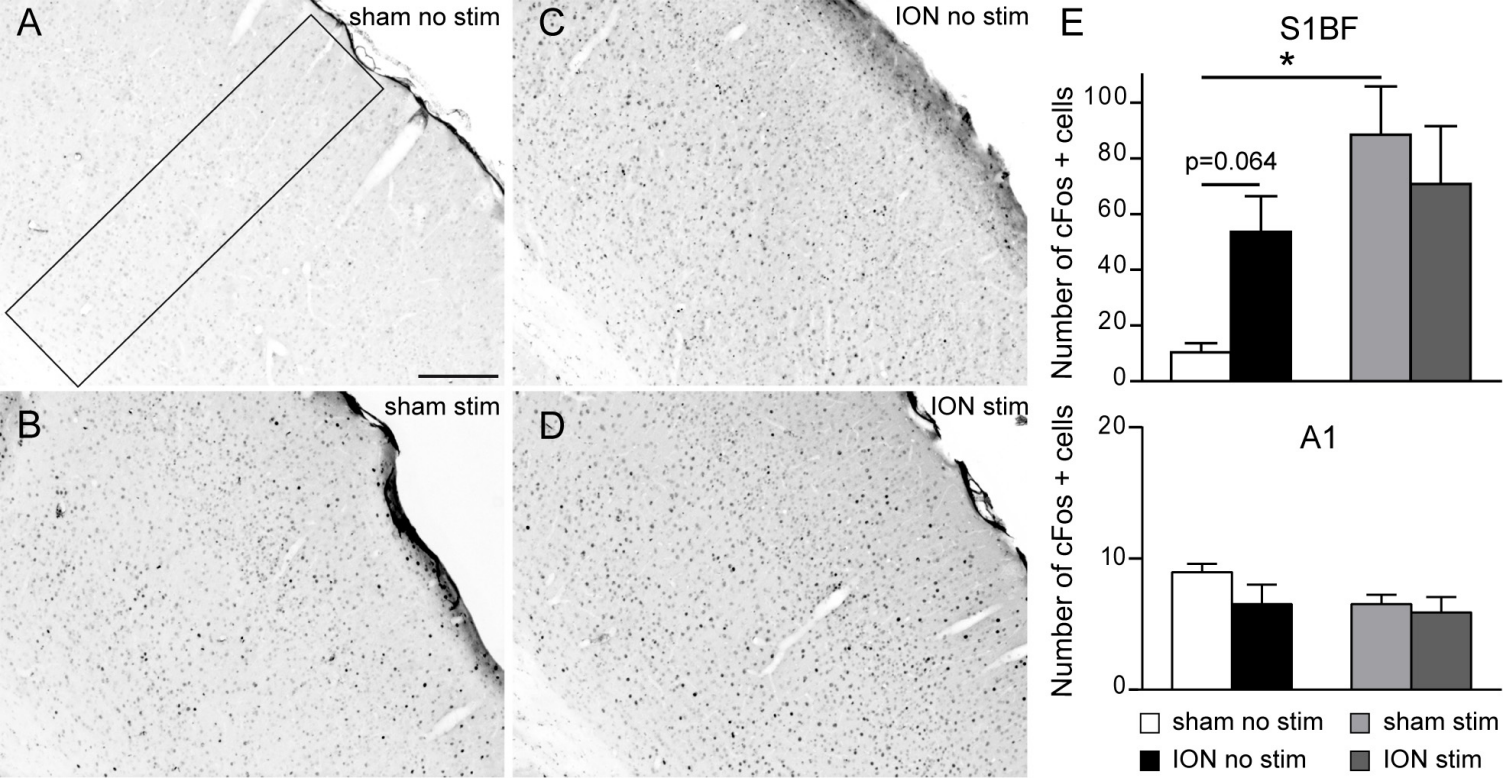
The absence of apparent cFos activation in response to sustained vibrissal stimulation in the cortex of ION constricted animals relies on an increased basal level of cFos in the S1BF cortex. Animals had all whiskers except one raw clipped and were left for two hours to explore an enriched environment. Following perfusion, the number of cFos positive profiles was analysed in the S1BF and primary auditory (A1) cortices. A-D: Representative examples of cFos staining observed in the contralateral side of the S1BF in non-stimulated sham (A), stimulated sham (B), non-stimulated ION (C) and stimulated ION (D) mice. Scale bar = 200 μm. E: Quantification of cFos-positive cells in the S1BF (top) and A1cortex in sham and ION mice 1 week post-surgery, after no stimulation or following 2 hours of natural vibrissal stimulation. Stimulation (Stim) induced a statistically significant upregulation of cFos in sham animals (p = 0.003) in the S1BF, although there was no difference in ION animals (p = 0.44). However, nerve ligation induced the upregulation of cFos in the S1BF of ION animals after no stimulation (p = 0.064). There was no change in the number of cFos positive cells in the A1 between all animals groups (bottom panel). Scale bars = 200 μm (n = 5 sham after no stimulation, n = 4 ION after no stimulation, n = 4 sham after stimulation, n = 5 ION after stimulation). Data are expressed as means ± SEM.

Therefore, in order to investigate whether the S1BF cortex of ION animals was subjected to enhanced neuronal activity, we examined the number of cells that were immunopositive for the proto-oncogene cFos in both sham and ION animals in two conditions: without any prior sensory stimulation (basal levels); and following sustained whisker stimulation for two hours (stimulated). In the absence of any stimulation, we observed an increase in the number of c-Fos-positive neurons in ION-ligated mice in the contralateral side of the S1BF cortex as compared to sham animals (Fig 4A–4C–4E), although this observation was not statistically significant (p = 0.064). As expected [35] after two hours of exploration in a stimulus-rich environment, the number of cFos-positive neurons increased significantly in the barrel field cortex of sham animals, compared to the basal condition (Fig 4A and 4B–4E; p<0.05). Interestingly, no difference between basal condition and stimulus-rich environment was observed in the ION-ligated group (Fig 4B–D and 4E), suggesting that the basal level of neuronal activity is increased in the S1BF of ION mice and can hardly be further increased by sensory (whisker) stimulation. These results suggest that there is an increased basal level of neuronal activity in S1BF cortical activity following sensory stimulation.

### ION ligation induces plasticity in different cortical areas

**Fig 5:**
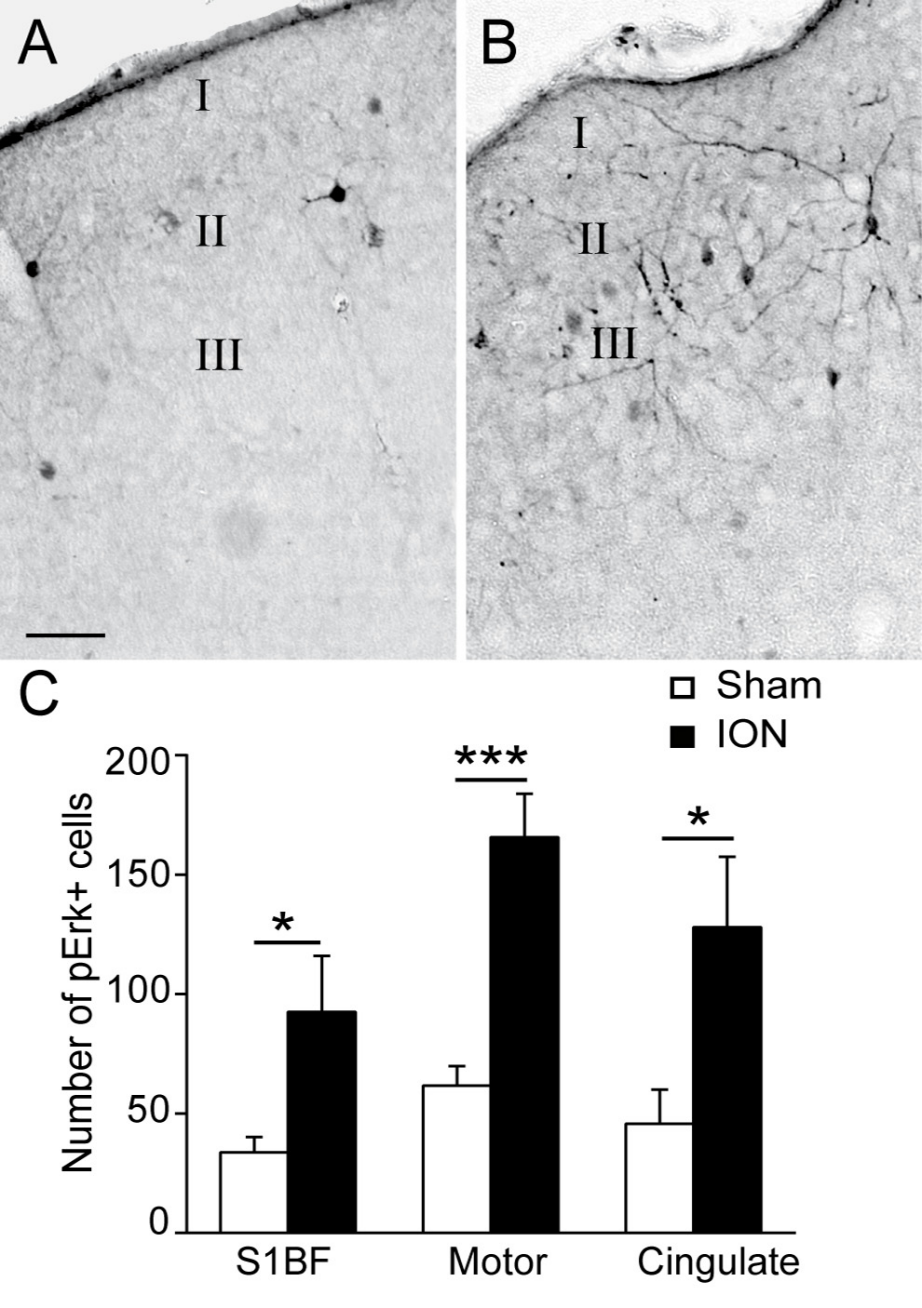
ION ligation induces the upregulation of p-Erk in different cortical areas. A-B: Representative examples of p-Erk staining in the barrel field cortex (S1BF) in sham (A) and ION (B) mice 5 weeks after the lesion/sham operation. Scale bars = 50μm. C: Quantification of the numbers of p-Erk-labelled cells in the S1BF, motor and cingulate cortices. ION ligation induced the upregulation of p-Erk+ cells. (F_1,26_ = 29.571; p<0.001, n = 6 sham, n = 5 ION)

To investigate the cortical pathophysiological changes occurring in our model, we next examined fluctuations in Erk phosphorylation as a marker of neuronal activity [43]. Acute and chronic pains have multiple components and numerous studies have described networks of brain areas activated by experimental pain. While some brain areas are rather involved in the sensory discriminative aspect of pain (lateral thalamus, S1 and motor cortex), others such as the cingulate and anterior insulate are rather involved in the affective-emotional aspect of pain [44–47]. We and others have also shown important changes in the anterior cingulate cortex associated with chronic inflammatory or neuropathic pain [48]. Consequently, our quantitative analysis of the number of p-Erk-positive cells was performed in the S1BF, motor and cingulate cortices 5 weeks post-injury. As previously observed by other teams, p-Erk cells are mostly located in pyramidal neurons in laminae II-III and laminae V and VI [49]. These results indicate that the number of p-Erk-positive cells was significantly increased in ION mice (Fig 5B) as compared to the sham group (Fig 5A) in the 3 structures (Fig 5C; p<0.05, p<0.001 and p<0.05 in the S1BF, motor and cingulate cortices, respectively).

**Fig 6:**
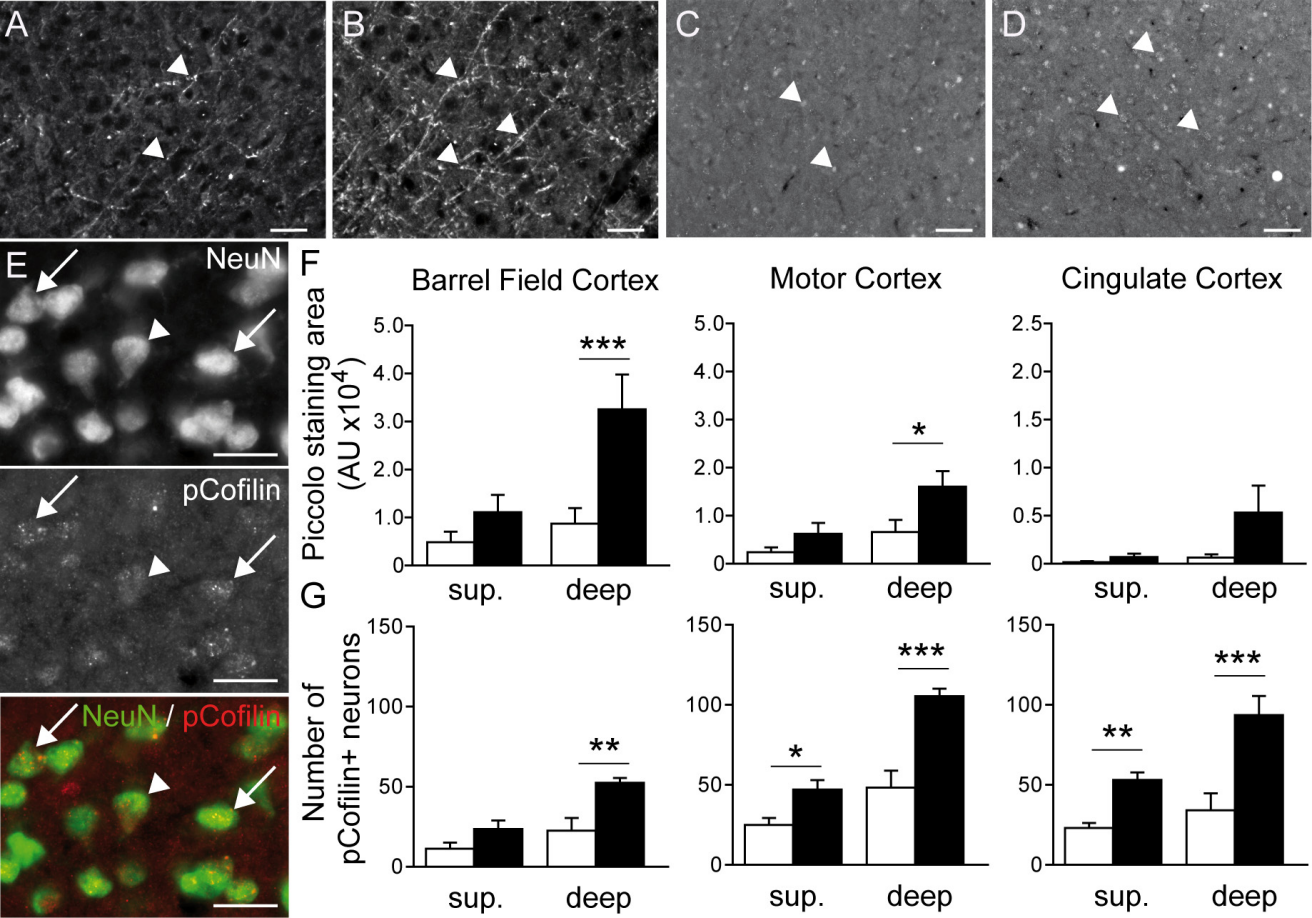
ION ligation induces synaptic plasticity in the S1BF, motor and cingulate cortices. A-B: Representative examples of Piccolo staining (arrow heads) in the deep laminae of the S1BF cortex in sham (A) and ION (B) mice 5 weeks after surgery. Scale bar = 50μm. C-D: Representative examples of p-Cofilin staining (arrow heads) in the deep laminae of the S1BF cortex in sham (C) and ION (D) mice. Scale bar = 50μm. E: Double immunostaining showing NeuN (green)/p-Cofilin (red) positive neurons in the S1BF (arrows). The arrow head point at a NeuN positive neuron negative for pcofilin. Scale bar = 25μm. F: Quantification of Piccolo staining in the S1BF, motor and cingulate cortices in deep and superficial laminae. ION ligation induced the upregulation of Piccolo staining in deep laminae in the S1BF and the motor cortex. Data are expressed as means ± SEM. *p<0.05 and **p<0.01 ***p<0.001 *vs*. sham mice, (n = 5 sham, n = 5 ION).

Based on these results, we focused on how this exacerbated cortical activity could be linked to neuronal plasticity mechanisms. Global changes of activity in neuronal networks are likely to induce homeostatic adaptations of synaptic strengths, which involve functional remodelling of both presynaptic and postsynaptic sites [49]. To measure presynaptic adaptations, we studied the expression of the Piccolo protein (Fig 6A and 6B), which is a component of the presynaptic cytoskeletal matrix assembled at the active zone of neurotransmitter release [50]and has been shown to be regulated upon homeostatic adaptation to network activity [51][52].

Piccolo expression was quantified by measuring the fluorescence staining area in the S1BF, motor and cingulate cortices (Fig 6F). We separated the analysis into two parts: the superficial layers including layers II through IV, and the deep layers including layers V and VI. Compared to the sham mice, the mean fluorescent staining area was significantly increased in the deep layers of the barrel field and motor cortices in ION mice (Fig 6F; p<0.001 and p<0.05, respectively). We also observed a non-significant increase in the mean fluorescent staining area in the deep layers of the cingulate cortex (Fig 6F).

Postsynaptic modifications were examined by measuring p-Cofilin expression (Fig 6C and 6D). Cofilin is a protein expressed in the postsynaptic compartment that increases the turnover of F-actin by severing its filaments, and it has also been shown to regulate immature spine structure [53][54]. The number of p-Cofilin–positive neurons was significantly increased in the deep layers of the barrel field, motor and cingulate cortices in ION mice (Fig 6G; p<0.01 and p<0.001). The ION mice also showed an increased number of p-Cofilin-positive neurons in the superficial layers of the motor and cingulate cortices (Fig 6G; p<0.05 and p<0.01, respectively).

## Discussion

We combined experimental approaches ranging from immunohistochemistry to *in vivo* functional imaging in order to study the neuronal correlates of orofacial neuropathic pain in the mouse cerebral cortex. Our results show that the motor, cingulate and barrel field cortices are the sites of p-ERK over-expression, a surrogate marker of neuronal activation, and over-expression of markers of active pre- and post-synaptic elements, suggesting an enhanced synaptic plasticity. These results provide a unique opportunity to increase our understanding of the molecular mechanisms underlying long-lasting cortical plasticity associated with persistent neuropathic pain.

### Ligation of the infraorbitral nerve induces long-lasting mechanical hyperalgesia

Traditionally, assessing trigeminal nerve-mediated nociceptive responses has been performed using methods that quantify brain stem-dependent behaviours (*e*.*g*. withdrawal responses or grooming) [55][28,56,57], in which the unlearned behaviours are elicited by mechanical stimulations using von Frey filaments [28] or thermal stimulation [30]. Such non-operant assessments may be relatively easy to execute; however, they evaluate innate behaviours that do not reveal cerebral processing of nociception. In this study we sought a more suitable approach, and used an orofacial operant test system. These types of pain tests employ a conflict paradigm that allows animals to make a choice between receiving a positive reward and escaping an aversive stimulus. For this reason, operant behavioural responses are not simple reflexive responses; rather, they should be considered as better indicators of pain in comparison to classical behavioural tests. Although operant behavioural responses are time-consuming (since they require several weeks of habituation), they are a better measure of spontaneous pain and their analysis can be fully automatized (therefore making them experimenter-independent). Orofacial operant tests have been used to characterize thermal (either cold [58,59] or hot temperatures [58,60–62]) and mechanical orofacial hypersensitivity [59,61]. Here, we have convincingly shown that mice experience long-lasting mechanical hyperalgesia following ION ligation, as previously reported in rat models [28].

### ION ligation exacerbates cortical activity

As previously described, ION ligation induced upregulation of ATF3, a marker of nerve injury [35,63–65] in TG neurons. This type of nerve injury is known to affect excitability in the sensory cortex [66]. Using *in vivo* intrinsic imaging, we observed that ION ligation induced a decreased haemodynamic response to vibrissae stimulation in the S1BF cortex. This result is consistent with a reduced haemodynamic responsiveness of the primary sensory cortex previously reported in a model of chronic stress and triggered by enhanced iNOS and decreased nNOS and HO-2 expression [67]. However our result was unexpected, since a previous voltage-sensitive dye imaging study had described an increased neuronal response in the primary somatosensory cortex of sciatic nerve-ligated rats, with an enlarged area of activation following electrical stimulation of the hind paw [41,68]. An increase in S1 responsiveness to cold stimuli (acetone applications) has also been observed in sciatic nerve-ligated rats by fMRI [42].

We further investigated this result by using cFos as a marker of neuronal activity. It was previously demonstrated that the increased neuronal expression of cFos is linked to higher neuronal activity in many neuronal networks, including the nociceptive pathways [69]. We observed that peripheral neuropathy induced by ION ligation is associated with an increase in cFos expression in the S1BF cortex under control conditions (although not significantly). Most strikingly, whisker stimulation *via* exposure to an enriched environment was not associated with increased cFos immunoreactivity in ION-ligated mice, in contrast to the sham-operated animals.

These results suggest that ION ligation may be at the origin of an enhanced and sustained activity in the trigeminal fibres [70,71] and/or an enhanced activation of thalamo-cortical connections, resulting in a postsynaptic enhanced neuronal activation of barrel field neurons. This increase in basal neuronal activity seems to preclude normal responses to tactile sensory input. Previous studies on sciatic nerve-ligated rats have reported a similar increase in cortical basal activity by monitoring either metabolic or vascular correlates of neuronal activity [71,72]. This hyperactivity was observed not only in the primary somatosensory cortex but also in other brain regions (*e*.*g*. the amygdala, the habenular complex, many thalamic nuclei, and the cingulate and retrosplenial cortices).

### ION ligation induces long-term cortical plasticity

We observed enhanced p-Erk immunoreactivity in cortical areas after ION ligation, confirming the hypothesized increase in basal cortical activity. Erk phosphorylation is considered to be a marker of spinal neuronal activation after application of a noxious stimulation [73,74], and previous studies have shown that Erk may contribute to rapid synaptic potentiation and the regulation of neuronal excitability [75]. Furthermore, Erk phosphorylation could result from changes in the strength of synapses. We therefore decided to study long-term adaptations of cortical synapses after ION ligation.

Synaptic adaptations are observed by modulating both presynaptic and postsynaptic functions. Modifications to the probability of neurotransmitter release and synapse size have both been reported at the presynaptic level [76,77], whereas receptor abundance and composition can be modified at the postsynaptic level, as reported for AMPA receptors [78–80]. Any of these parameters could participate in the cortical hyperactivity observed in our model. Neuropathic pain and memory processes exhibit adaptive mechanisms including long-term potentiation and central sensitization [3,81], and these mechanisms depend upon synaptic plasticity.

Prolonged activity depletion induces a significant downregulation in the cellular expression levels of presynaptic scaffolding proteins including Bassoon, RIM, synapsin and Piccolo [52]. The ADF/Cofilin complex may regulate synaptic strength at the postsynaptic compartment. Indeed, ADF/Cofilin phosphorylation and dephosphorylation have been associated with spine growth and shrinkage during LTP and LTD, respectively [82]. Moreover, the number of p-Cofilin-positive puncta is dependent on excitatory transmission [83,84] and use of lentiviral vectors and optogenetically labelled cofilin1 knocked-down-neurons showed that cofilin-1 mediated actin dynamics regulates functional cortical map plasticity in an input-specific mechanism [85]. These data, combined with the upregulation of Piccolo and p-Cofilin observed in our model, suggest the presence of functional and structural plasticity induced by the neuropathic state in diverse cortical areas. The upregulated number of synapses and/or the strength of these synapses may participate in the long-lasting sensory changes in neuropathic mice observed concomitantly with mechanical hyperalgesia. Kim and Nabekura reported a rapid synaptic remodelling following peripheral nerve injury, accompanied by the formation of new spines in the early phase of neuropathic pain [86]. Our results suggest that this remodelling persists into the late phase of neuropathic pain, as revealed by the increased expression of both pre- and postsynaptic markers.

### ION ligation-induced cortical plasticity is layer-specific

Our results revealed significant synaptic modifications within cortical deep layers, particularly in the S1BF cortex, where we observed the upregulation of Piccolo and p-Cofilin within layers V and VI. We principally observed the upregulation of p-Cofilin in the deep layers of the motor and cingulate cortices, but also in the superficial layers. One previous report has shown, in the S1 cortical representation of the hind limb in naive rats, that neurons exclusively driven by noxious stimulation are located preferentially in laminae Va, Vb, VIa and VIb, *i*.*e*. in the deeper laminae [66]. Similarly, a recent study based on cFos immunoreactivity reported that noxious stimuli in naive mice seem to preferentially activate the subgranular layer Va [33]. Single-unit extracellular recordings in ION rats have revealed reduced background activity of whisker neurons (compared to naive rats) in laminae II-III and IV, accompanied by strongly increased activity in lamina VIa [33,68]. Altogether, these results suggest that the subgranular layers might be particularly involved in the processing of noxious stimuli. In light of our observations, they also appear to be involved in synaptic plasticity mechanisms accompanying trigeminal neuropathic pain.

As in the sensory cortices, where layer VI appears to influence the activity of thalamic neurons that relay sensory information [87,88], the upregulation of active synapses after nerve ligation in deep layers suggests that neuropathic pain may be associated with reorganization at the thalamo-cortical level. Previous studies have demonstrated that neuropathic pain conditions are associated with changes in thalamic anatomy and biochemistry [21,89,90], suggesting that firing patterns within thalamo-cortical loops are altered in the neuropathic pain state.

These results suggest a novel conceptual framework for the comprehension of chronic-pain induced cortical plasticity, by showing that long-lasting orofacial neuropathic pain is associated with exacerbated neuronal activity and synaptic plasticity. The resulting over-enhanced synaptic strength, putatively upregulated from it's physiological optimum, may explain both exacerbated sensitivity (allodynia) and loss of performance in fine-textured sensory discrimination tasks, both reported in chronic pain patients [44]. Consequently, controlled therapeutic regulation of structural synaptic plasticity in chronic pain may represent a novel and original clinical target, to be validated in future pre-clinical studies.

## Supporting Information

S1 Dataset Mini dataset presenting in different tabs individual results from Sham and ION treated animals. Each tab shows the results for the following items: ATF3 positive DRG neurons (Fig 1), behaviour (time spent drinking, which provides an indirect measure of mechanical hyperalgesia, Fig 2), intrinsic optical imaging (Fig 3), c-fos expression in the S1BF (Fig 4), and immunostainings quantifications in different cortical areas: p-ERK (Fig 5), Piccolo and p-Cofilin (Fig 6).
(XLSX)

S1 Table Table reporting the number of animals included in the initial experimental design, and the reasons for the exclusion of some of them during the course of the study. For instance, some animals were excluded from the imaging experiments when they lost some of their whiskers, or from the behavioural analysis if they were not drinking during the habituation sessions. Additionally, one mouse died from unknown reasons. The three bottom lines indicate the number (“N”) of animals included for each one of the following approaches (Behaviour, Imaging and Immnohistochemistry (“Immuno”)) at the different time points during of these experiments.
(XLSX)
